# Bilevel opposite direction ESP block with indwelling catheter in the management of severe lung cancer pain

**DOI:** 10.1016/j.inpm.2022.100144

**Published:** 2022-09-06

**Authors:** Ahmet Murat Yayik, Yunus Emre Karapinar, Habip Burak Ozgodek, Ibrahim Hakki Tor, Ali Ahiskalioglu

**Affiliations:** Department of Anaesthesiology and Reanimation, Ataturk University Faculty of Medicine, Erzurum, Turkey; Clinical Research, Development and Design Application and Research Center, Ataturk University School of Medicine, 25240, Erzurum, Turkey, Erzurum, Turkey; Department of Anaesthesiology and Reanimation, Ataturk University Faculty of Medicine, Erzurum, Turkey; Department of Anaesthesiology and Reanimation, Ataturk University Faculty of Medicine, Erzurum, Turkey; Department of Anesthesiology and Reanimation, University of Health Sciences, Erzurum Regional Training and Research Hospital, Erzurum, Turkey; Department of Anaesthesiology and Reanimation, Ataturk University Faculty of Medicine, Erzurum, Turkey; Clinical Research, Development and Design Application and Research Center, Ataturk University School of Medicine, 25240, Erzurum, Turkey

## Abstract

One of the biggest challenges faced by pain physicians is that cancer patients present with unrelieved pain despite multimodal drug regimes. Regional anesthesia methods and indwelling catheters become significant when pain control cannot be achieved with these regimens. Ultrasound-guided erector spinae plane (ESP) block has provided analgesia for acute postoperative and chronic cancer pain. This is a case of a 58-year-old male with no significant medical history prior to being diagnosed with lung cancer two years ago who is having severe pain in the entire hemithorax after the diagnosis. ESP block with the indwelling catheter was administered at two different levels in opposite directions, one from the T5 level in the caudal-cranial direction and one from the T7 in the cranial-caudal direction, provide adequate analgesia between T2-T12 dermatomes. Bilevel opposite direction ESP block with an indwelling catheter may result in better analgesia in oncologic patients where pain control cannot be achieved with opioids.

To the Editor,

In chronic cancer pain management, it is essential to apply step therapy ranging from paracetamol to potent opioids for pain control [1]. In cases of terminal cancer patients where pain control cannot be achieved despite strong opioids, regional anesthesia methods come to the forefront. As demonstrated in the case series of which 2 cases of neuropathic pain and 2 cases of acute postsurgical pain published by Forero et al., erector spinae plane block (ESPB) can be used successfully in the treatment of severe thoracic neuropathic pain [2].

ESPB has been used to control acute pain however [3], in this case report, we present a relatively more novel use of tunneled opposite direction ESPB in a patient with terminal lung cancer who had diffuse pain on the entire left hemithorax unresponsive to medical treatments*.* Written informed consent for all procedures and publication of data was obtained from the patient. In this letter, we report the case of a 58-year-old male with no significant medical history prior to being diagnosed with lung cancer 2 years ago. He was subsequently treated with chemotherapy but was experiencing severe thoracic pain for which he presented to our pain clinic. His laboratory findings did not show anything significant in terms of coagulation profile and other parameters. Although the patient was using dexketoprofen 100 mg/day iv, dexamethasone 8 mg/day iv, pregabalin 300 mg/day, and fentanyl transdermal patch 100 mcg/h during his hospitalization, the VAS (visual analog score) pain score was 9/10. The localization of the pain was unilateral in the T3-T12 region over the left hemithorax.

ESPB was performed with 30 ml of 0.25% bupivacaine at the T5 level as a test block to evaluate the block efficiency on the first day. In the dermatomal examination performed 30 minutes after the block, it was observed that anesthesia was provided between T2-T8 dermatomes on the left hemithorax. In the clinical follow-ups, it was observed that while effective analgesia (VAS≤3) was provided between T2-T8 dermatomes for 18 hours, severe pain continued in T8-T12 dermatomes. Subsequently, we planned to insert an ESPB catheter at the T5 level in a caudal-cranial direction and at the T7 level in a cranial-caudal direction in an attempt to effectively provide analgesia in the left hemithorax between T2-T12.

After sterile preparation, an USG (ultrasonography) probe (4–12 MHz) was placed parasagitally at the T5 vertebra level; the transverse process, erector spinae, and trapezius muscles were identified. After the local anesthetic injection, the lower fascia of the erector spina muscle was passed with an 18G tuohy needle. After confirmation of needle position using 2 ml of saline, 20 ml of 0.5% bupivacaine was injected. Next, a 22 G catheter was inserted through the tuohy needle into the same plane and then the catheter was tunneled under the skin. The process was repeated in the opposite direction in the T7 level (Fig. 1). 20 ml of 0.125% bupivacaine was administered every 8 hours through both catheters for three days during the hospitalization period. The same regimen was applied after discharge. His medical treatment was rearranged, and given the patient's improved analgesia, his fentanyl patch was discontinued. Catheter usage training was given to the patient and his family (he was taught about stopping injection when itching, tinnitus, perioral numbness, metallic taste occurs and to aspirate before boluses and stop when a bloody aspiration is seen) and he was discharged with the recommendation to use the catheter for pain control. The patient was called on a weekly basis to assess for analgesia efficacy as well as any concerns for catheter malfunction or adverse events. We planned to potentially replace the catheter in the event of patient-reported catheter malfunction or loss of analgesic effect. In our reported case, the catheter was deemed to be functioning properly and thus there was no need for replacement. The catheter was used effectively and provided analgesia (VAS <4) for 28 days after discharge. After 28 days of successful usage of catheter, the patient passed away.

**Fig. 1:**
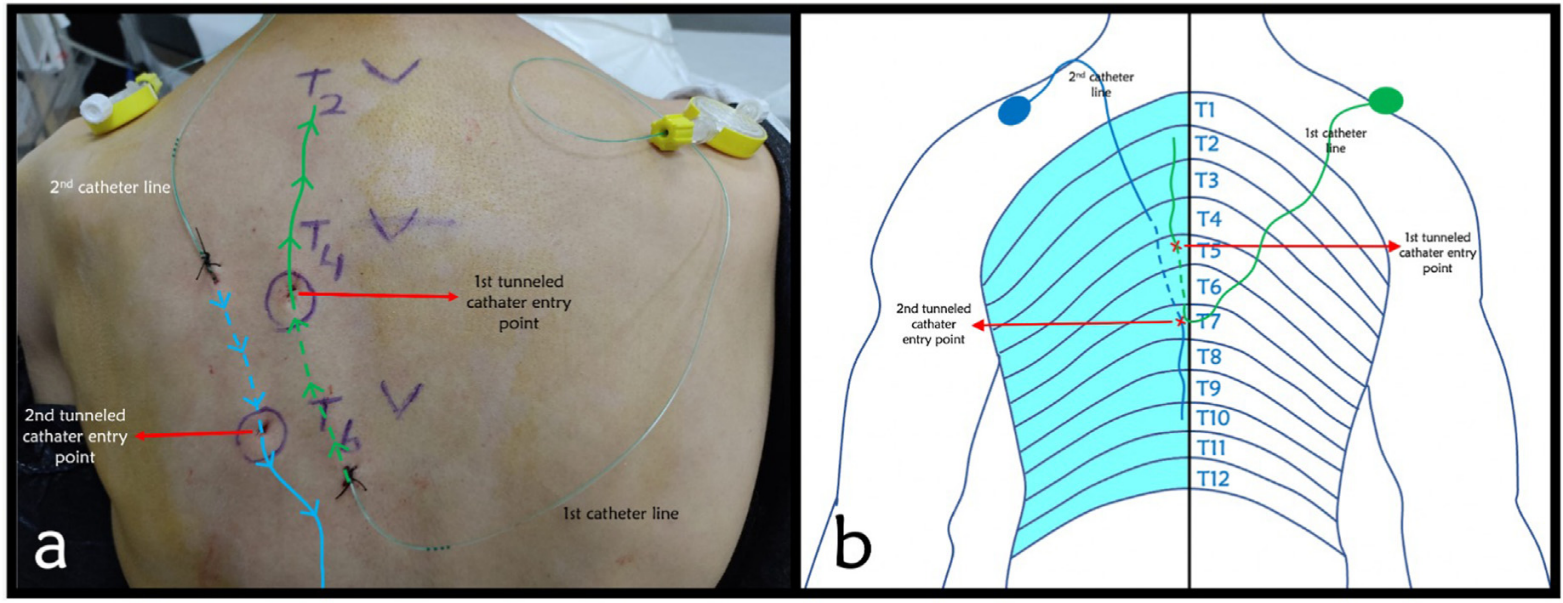
a) The blue line represents the craniocaudal, the green line the caudocranial inserted catheter. The dashed lines show the tunneled part of the catheters (directions of the catheters are marked by arrows on figure) b) Represents dermatomal distribution of pain and the levels of catheters placed with entry points and tunneled parts. (Dashed lines show the tunneled parts).. (For interpretation of the references to colour in this figure legend, the reader is referred to the Web version of this article.)

To conclude, thoracic epidural analgesia, thoracic paravertebral block and thoracic ESPB can be used as alternative analgesia methods for thoracic neuropathic pains. However, it is generally known that hypotension, urinary retention, and postoperative nausea and vomiting are less frequently seen with thoracic paravertebral block and thoracic ESPB when compared with thoracic epidural analgesia [4]. ESPB generally has a lower complication rate compared to thoracic epidural analgesia when used as either a single injection or a continuous catheter-based infusion treatment. It is a question that how long nerve block catheters can safely be used and whether continuous infusions or intermittent boluses for fascial plan blocks are more effective and both are debated topics [5], in our clinical experience, we have observed that intermittent boluses are more effective than continuous infusions. Also, it was not possible for us to use a PCA (Patient-Controlled Analgesia) infusion pump for outpatient control. This experience and the facility utilities forced us to use an indwelling catheter with intermittent boluses rather than continuous infusions.

In addition, long-term usage of neuraxial catheter is limited due to possible destructive complications such as meningitis and epidural abscess whereas ESPB catheter is safer for continuous usage. Theory behind this idea emerges from its superficial placement and being away from the neuraxial structures. In addition, based on risk-benefit analysis, obstacles like anticoagulation can be overcome [6]. To conclude, ESPB is widely used for both postoperative analgesia and for surgical anesthesia in surgical procedures with bilevel high volume injections which could increase the block success. Bi-level opposite direction ESPB may have an improved effect for targeting wide dermatomal analgesia compared to conventional single-level ESPB, as previously mentioned in the literature [7,8]. In this case report we want to emphasize that ultrasound guided ESPB catheters can be considered as an alternative treatment method for unilateral regional oncological pain cases that do not respond to medical treatment.

## Funding

The authors have no sources of funding to declare for this manuscript.

## Declaration of competing interest

The authors declare that they have no known competing financial interests or personal relationships that could have appeared to influence the work reported in this paper.
